# High Fat Diet-Induced Obesity Dysregulates Splenic B Cell Mitochondrial Activity

**DOI:** 10.3390/nu15224807

**Published:** 2023-11-17

**Authors:** Anandita Pal, Chien-Te Lin, Ilya Boykov, Emily Benson, Grahame Kidd, Kelsey H. Fisher-Wellman, P. Darrell Neufer, Saame Raza Shaikh

**Affiliations:** 1 Department of Nutrition, Gillings School of Global Public Health and School of Medicine, The University of North Carolina at Chapel Hill, Chapel Hill, NC 27599, USA; ananditapal9@gmail.com; 2 East Carolina Diabetes and Obesity Institute, Brody School of Medicine, East Carolina University, Greenville, NC 27834, USA; linch@ecu.edu (C.-T.L.); boykovi10@students.ecu.edu (I.B.); fisherwellmank17@ecu.edu (K.H.F.-W.); 3 Department of Physiology, Brody School of Medicine, East Carolina University, Greenville, NC 27834, USA; 4 3D-EM Ultrastructural Imaging and Computation Core, Lerner Research Institute, Cleveland Clinic, Cleveland, OH 44106, USA; bensone2@ccf.org (E.B.); kiddg@ccf.org (G.K.); 5 Department of Biochemistry and Molecular Biology, Brody School of Medicine, East Carolina University, Greenville, NC 27834, USA

**Keywords:** high fat diet, B cells, obesity, mitochondria, proteome

## Abstract

Diet-induced obesity impairs mitochondrial respiratory responses in tissues that are highly metabolically active, such as the heart. However, less is known about the impact of obesity on the respiratory activity of specific cell types, such as splenic B cells. B cells are of relevance, as they play functional roles in obesity-induced insulin resistance, inflammation, and responses to infection. Here, we tested the hypothesis that high-fat-diet (HFD)-induced obesity could impair the mitochondrial respiration of intact and permeabilized splenic CD19+ B cells isolated from C57BL/6J mice and activated ex vivo with lipopolysaccharide (LPS). High-resolution respirometry was used with intact and permeabilized cells. To reveal potential mechanistic targets by which HFD-induced obesity dysregulates B cell mitochondria, we conducted proteomic analyses and 3D serial block face scanning electron microscopy (SBFEM). High-resolution respirometry revealed that intact LPS-stimulated B cells of obese mice, relative to controls, displayed lower ATP-linked, as well as maximal uncoupled, respiration. To directly investigate mitochondrial function, we used permeabilized LPS-stimulated B cells, which displayed increased H_2_O_2_ emission and production with obesity. We also examined oxidative phosphorylation efficiency simultaneously, which revealed that oxygen consumption and ATP production were decreased in LPS-stimulated B cells with obesity relative to controls. Despite minimal changes in total respiratory complex abundance, in LPS-stimulated B cells of obese mice, three of the top ten most downregulated proteins were all accessory subunits of respiratory complex I. SBFEM showed that B cells of obese mice, compared to controls, underwent no change in mitochondrial cristae integrity but displayed increased mitochondrial volume that was linked to bioenergetic function. Collectively, these results establish a proof of concept that HFD-induced obesity dysregulates the mitochondrial bioenergetic metabolism of activated splenic B cells.

## 1. Introduction

Obesity is well established to impair the mitochondrial respiratory function of metabolically active tissues, such as the heart [1]. Mitochondria are of interest, as they are increasingly recognized to be targets for therapeutic development, given their essential role in maintaining cellular bioenergetics and controlling cell death cascades [2]. Previous research using differing models of obesity or ischemic injury has revealed that mitochondrial respiratory function is impaired through a wide range of biochemical and biophysical mechanisms [3,4]. Less is known about whether diet-induced obesity also impacts the mitochondrial respiratory activity of other tissues and associated cell types. Herein, we focused on the impact of high-fat-diet (HFD)-induced obesity on murine splenic B cell respiratory activity. 

Studies across human and murine models have revealed that obesity is associated with dysregulated B cell development, cytokine secretion, class switching, and antibody production [5]. This dysregulation may contribute to the onset and progression of chronic inflammation, glucose dysregulation, and insulin resistance [6,7,8,9]. Notably, weight gain upon metabolic overload leads to the enrichment of select B cell subsets in white adipose tissue, which can drive inflammation that is linked to impaired glucose uptake [9]. Furthermore, impaired B cell functional responses are also a contributing factor toward susceptibility to infections and poor responsiveness to vaccinations [10,11]. For instance, antibody production is decreased upon influenza infection in obese mice, which may contribute to increased morbidity and mortality [12]. Taken together, there is a need to establish whether diet-induced obesity drives dysregulation at the level of B cell mitochondrial activity. 

The primary objective of this study was to determine whether HFD-induced obesity could dysregulate key aspects of mitochondrial respiratory function of isolated splenic CD19+ B cells activated with lipopolysaccharide (LPS). We focused on splenic B cells, as they are highly abundant for high-resolution respirometry studies. Importantly, the spleen plays a critical role in differing functions for B cells, such as the generation of memory. The second objective was to take the first steps toward understanding how HFD-induced obesity may impair B cell respiratory function. For these experiments, we relied on proteomic analyses and serial block face scanning electron microscopy.

## 2. Materials and Methods

Mice, diets, and metabolic phenotyping: C57BL/6J male mice with a starting age of 5 weeks were placed on a lean mouse chow or high-fat (60% lard) diets (Research Diets, New Brunswick, NJ, USA) for 12 weeks, as previously described [13]. Body weight measurements were collected weekly. The metabolic state of the animals was characterized by an intraperitoneal glucose tolerance test (IPGTT) after a 5 h fast at week 12 of the diet treatment. The glucose levels during the IPGTT were measured with a glucometer, and insulin levels were assayed with an ELISA (Crystal Chem, Elk Grove Village, IL, USA) [14]. To determine a baseline, glucose levels were measured with a glucometer 15 min before glucose injection at time 0. 

B cell isolation and activation. Spleens were harvested upon the sacrifice of the lean and obese mice, and CD19+ B cells were isolated using a B cell isolation kit (Invitrogen, Waltham, MA, USA), as previously described [15]. The 3E6 cells per mL per diet per mouse were activated with 1 μg/mL LPS (Sigma Aldrich, St. Louis, MO, USA) for 24 h, followed by another live cell counting and the resuspension to the desired cell concentration in either buffer Z or DEME media (HCO_3_ free). 

High-resolution respirometry to measure basal, ATP-linked, maximal, and non-mitochondrial oxygen consumption. Mitochondria respiration assays were performed in a high-resolution respirometer (O2K, Oroboros, Tyrol, Austria) to determine the steady state rate of oxygen consumption (JO_2_, i.e., e–flow through an electron transport system). The 3E6 intact cells per mL in DEME media (HCO_3_-free) were loaded into an O2K chamber (1 mL) at 37 °C with continuous stirring. Then, 1 mM of pyruvate, 10 mM of glucose, and 4 mM glutamine were added to ensure a sufficient substrate for active respiration (i.e., basal plus ATP synthesis). Oligomycin (1 μM) was then used to inhibit the ATP synthesis component, followed by the uncoupler FCCP (carbonyl cyanide-4 (trifluoromethoxy) phenylhydrazone) (1.5 μM) to determine the maximal respiratory capacity. Rotenone (2 μM) and Antimycin A (2.5 μM) were added to inhibit complexes I and III, respectively. 

The simultaneous measurement of mitochondrial O_2_ consumption and ATP production: ATP/O analysis was performed as previously described [16,17,18] to directly measure OXPHOS efficiency (ATP production in relation to O_2_ consumption or the ATP/O ratio). Briefly, the rates of mitochondrial ATP synthesis (JATP) and JO_2_ were measured simultaneously at three clamped steady state rates using a custom-designed system that couples high-resolution respirometry with a fluorescence-based enzyme-linked assay for detecting JO_2_ and JATP, respectively. The 3E6 cells per mL in buffer (5 mM of creatine, 105 mM of K-MES, 30 mM of KCl, 1 mM of EGTA, 10 mM of K_2_HPO_4_, 5 mM of MgCl_2_-6H_2_O, and 0.5 mg/mL of BSA, pH 7.1) were loaded into the O2K chamber (1.3 mL, 37 °C, and continuous stirring). The cells were permeabilized with 10 μg/mL of saponin for 10 min in the assay chamber, and a baseline was obtained. Then, 0.1 mM of AP5A was added to inhibit adenylate kinase. The cells were then energized with multiple mitochondrial substrate combinations ((PMGSO: 5 mM pyruvate (P) + 0.5 mM malate (M) + 5 mM glutamate (G) + 10 mM succinate (S) + 0.2 mM octanoyl-carnitine (O)), followed by 200 μM of ADP. NADPH auto-fluorescence was recorded, and a standard curve was used to calculate the ATP concentration and rate of change in the chamber.

Mitochondrial H_2_O_2_ emission and production rate (JH_2_O_2_): JH_2_O_2_ was measured fluorometrically, as previously described [17,19,20], with a slight modification. The cells were resuspended to 3E6 cells per mL in Buffer Z that was supplemented with 10 μM of AUR, 2 U/mL of HRP, and 25 U/mL of CuZn-SOD. After loading into a cuvette (0.2 mL, 37 °C, and continuous stirring), the cells were permeabilized using 10 μg/mL of saponin for 10 min in a cuvette and obtaining a steady background. The mitochondria were then energized with multiple mitochondrial substrate combinations of PMGSO to trigger maximal JH_2_O_2_ emission. Then, 0.1 μM of auranofin (AF) and 0.1 mM of 1,3-bis(2-chloroethyl)-1-nitrosourea (BCNU, Carmustine) were further added. AF, at 0.1 μM, inhibits thioredoxin reductase, and BCNU inhibits glutathione reductase. Together, AF and BCNU block the main matrix redox buffering circuits and reveal the mitochondria H_2_O_2_ production rate.

Sample preparation for proteomic analyses: The preparation of cells for label-free proteomics analysis was performed as previously described [21,22]. The cell pellets were lysed in urea lysis buffer (8 M of urea in 40 mM of Tris, 30 mM of NaCl, 1 mM of CaCl_2_, and 1 x complete ULTRA mini EDTA-free protease inhibitor tablet; pH = 8.0). Following two freeze–thaw cycles, the samples were sonicated with a probe sonicator in three 5 s bursts (Q Sonica #CL-188; amplitude of 30). The samples were centrifuged at 10,000× *g* for 10 min at 4 °C, and the protein concentration was determined via BCA. Equal protein amounts were reduced with 5 mM of DTT at 37 °C for 30 min and then alkylated with 15 mM of iodoacetamide for 30 min in the dark. Unreacted iodoacetamide was quenched with DTT (15 mM). Reduction and alkylation reactions were carried out at room temperature. Initial digestion was performed with Lys C (1:100 *w*:*w*) for 4 h at 32 °C. Following dilution to 1.5 M of urea with 40 mM of Tris (pH = 8.0), 30 mM of NaCl, and 1 mM of CaCl_2_, the samples were digested overnight with sequencing-grade trypsin (50:1 *w*/*w*) at 32 °C. The samples were acidified to 0.5% TFA and then centrifuged at 4000× *g* for 10 min at 4 °C. A supernatant containing soluble peptides was desalted, and then the eluate was frozen and subjected to speedvac vacuum concentration.

nLC-MS/MS for label-free proteomics: As previously described [21,22], peptides were resuspended in 0.1% formic acid, quantified (ThermoFisher Cat# 23275, Waltham, MA, USA), and then diluted to a final concentration of 0.25 µg/µL. The samples were subjected to nanoLC-MS/MS analysis using an UltiMate 3000 RSLCnano system (ThermoFisher) coupled with a Q Exactive Plus Hybrid Quadrupole-Orbitrap mass spectrometer (ThermoFisher) via a nanoelectrospray ionization source. For each injection, 4 µL (1 µg) of the sample was first trapped on an Acclaim PepMap 100 20 mm × 0.075 mm trapping column (ThermoFisher Cat# 164535; 5 μL/min at 98/2 *v*/*v* water/acetonitrile with 0.1% formic acid). Analytical separation was performed over a 95 min gradient (a flow rate of 250 nL/min) of 4–25% acetonitrile using a 2 µm EASY-Spray PepMap RSLC C18 75 µm × 250 mm column (ThermoFisher Cat# ES802A) with a column temperature of 45 °C. MS1 was performed at a 70,000 resolution with an AGC target of 3 × 10^6^ ions and a maximum injection time (IT) of 100 ms. MS2 spectra were collected via the data-dependent acquisition (DDA) of the top 15 most abundant precursor ions with a charge greater than 1 per MS1 scan, with dynamic exclusion enabled for 20 s. The precursor ions’ isolation window was 1.5 *m*/*z*, and the normalized collision energy was 27. MS2 scans were performed at a 17,500 resolution, a maximum IT of 50 ms, and an AGC target of 1 × 105 ions.

Data analyses for label-free proteomics: As described previously [21,22], with some modification, Proteome Discoverer 2.2 (PDv2.2) was used for raw data analysis, with default search parameters including oxidation (15.995 Da on M) as a variable modification and carbamidomethyl (57.021 Da on C) as a fixed modification. The data were searched against the Uniprot Mus musculus reference proteome (Proteome ID: UP000000589), as well as the Human Mito Carta 2.0 database [23]. PSMs were filtered to a 1% FDR and grouped to unique peptides while maintaining a 1% FDR at the peptide level. Peptides were grouped to proteins using the rules of strict parsimony, and proteins were filtered to 1% FDR. Peptide quantification was performed using the MS1 precursor intensity. Imputation was performed via low-abundance resampling. As described previously [21,22,24], using only high-confidence master proteins, data were normalized to both the total protein amount and the total abundance of mitochondrial proteins, based on MitoCarta 2.0.

Volume EM–Serial blockface scanning EM imaging: B cell samples were prepared as described above, fixed in 4% paraformaldehyde and 2.5% glutaraldehyde in 0.1 M of an Na cacodylate buffer. The cells were gently pelleted and embedded in low-melt agarose; then, they were stained for volume EM using the ASP-1000 (Microscopy Innovations, Marshfield, WI, USA), as previously described for tissue samples [25]. The pellets were trimmed from the resin and mounted on aluminum pins and coated with colloidal silver. Imaging was undertaken using both a VolumeScope II (ThermoFisher Scientific) system and a 3View in-chamber ultramicrotome (Gatan, Pleasanton, CA, USA) on a Sigma VP FE-SEM (Zeiss, Oberkochen, Germany). Fields of 30 × 30 µm were imaged at 4.5 nm/pixel (2.0 kV, a 30 µm aperture, and standard vacuum mode) with 50–65 nm slices and 300–500 slices in z-depth. 

Image analyses: The image stacks contained 30–50 B cells from each sample. From each sample stack, five or more whole cells were cropped as substacks (additional details are described below) and used for analysis. For fractional volume determination, the mitochondria, cell membrane, and nucleus were segmented in slices 1 µm apart using a combination of manual and deep learning annotation. Binary images (masks) of each segmented area were generated, and slab volumes (segmented area × 1 µm thickness) were summed across the stack. The cell fraction occupied by the mitochondria was determined with and without the nucleus. A profile of the mitochondrial shape and size in each cell was generated from 3–6 mitochondria that were chosen randomly in each cell. Each was segmented manually across all slices of the 60 nm stack in which it occurred. Binary masks were generated, and the volume of each mitochondrion was generated as above. Length and aspect ratios were assessed from the 3D bounding boxes delineating each mitochondrion. These data were used to assess whether the mitochondria were predominantly elongated, predominantly small and spherical, large and spherical (i.e., swollen), or a healthy mix. The cristae ultrastructure was measured in three ways. For the qualitative observation of cristae, mitochondria in 3–5 contiguous 60 nm slices were segmented, binary masks generated, and the mask used to remove all other elements of the image, and then 3D reconstructions were generated by projecting the EM image using ImageJ or Dragonfly. The mitochondrial membrane-only volume was calculated via the density-based segmentation of the cristae and inner and outer mitochondrial membranes in 1 µm separated slices. Inter-cristae spacing was determined in 1µm spaced slices. A line plot along the long axis of the mitochondrion was used to identify intensity peaks that represented each crista. The average spacing for each mitochondrial profile was generated as the number of peaks divided by the length of the line. Statistical analyses were undertaken using R (R 3.6.3 (2000-02-29)).

Statistical analyses: All data sets were confirmed to display normalized distributions using a Kolmogorov–Smirnov test. The data were analyzed with an unpaired Student’s *t*-test. Body weight and insulin/glucose data that were collected as a function of time were analyzed with a two-way ANOVA, followed by a post hoc Sidak multiple-comparison test. *p* values of less than 0.05 were considered significant. 

## 3. Results

**Figure 1:**
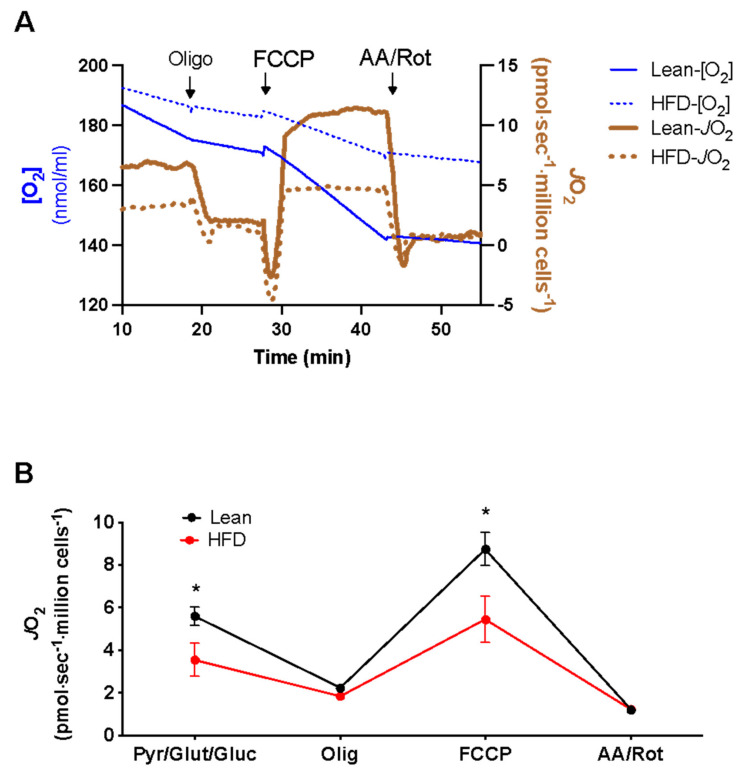
**The** CD19+ B cells isolated from HFD-induced obese mice display lower basal, ATP - linked, and uncoupled respiration. The C57BL/6J mice were fed a control lean or high-fat diet followed by the isolation of splenic CD19+ B cells that were activated for 24 h in a culture with LPS. (**A**) Raw O_2_K traces for O_2_ and JO_2_ in response to a standard respiration protocol, as described in the Materials and Methods section. (**B**) JO_2_ in response to pyruvate/glutamine/glucose, oligomycin, FCCP, and antimycin A/rotenone. Data are averages ± SEMs from seven independent mice experiments. * *p* < 0.05.

We first ensured that the mice were metabolically impaired compared to the lean controls. Their body weights were significantly increased within four weeks of the HFD intervention relative to the lean controls (Supplementary Figure S1A). Glucose and insulin values were also measured in response to an IPGTT. As expected, the concentration of glucose (Supplementary Figure S1B) and insulin (Supplementary Figure S1C) was elevated with mice on an HFD compared to the lean controls. Respiratory function was then analyzed using intact splenic CD19+ B cells that were activated ex vivo with LPS and then subjected to a standard respiration protocol for intact cells in a suspension using the high-resolution respirometer. Raw traces of the results are presented in Figure 1A. The basal oxygen consumption rate (JO_2_) was significantly lower in the splenic B cells of the obese mice relative to the lean animals (Figure 1B). The addition of oligomycin (an inhibitor of ATP synthase) reduced JO_2_ to identical values, indicating that ATP-linked JO_2_ (basal minus oligo rates) accounted for the lower basal oxygen consumption rate in intact cells of obese mice. The maximal uncoupled JO_2_ was also significantly lower for the obese mice compared to the lean mice, as measured in response to FCCP (an uncoupler), indicating a lower respiratory reserve and maximal capacity in the B cells of obese mice (Figure 1B). These data suggest that HFD-induced obesity decreases B cell mitochondrial respiratory function. Finally, we measured non-mitochondrial respiration using a combination of rotenone (inhibits complex I) and antimycin A (inhibits complex III). There was no difference in JO_2_ with these inhibitors between the lean and obese animals.

**Figure 2:**
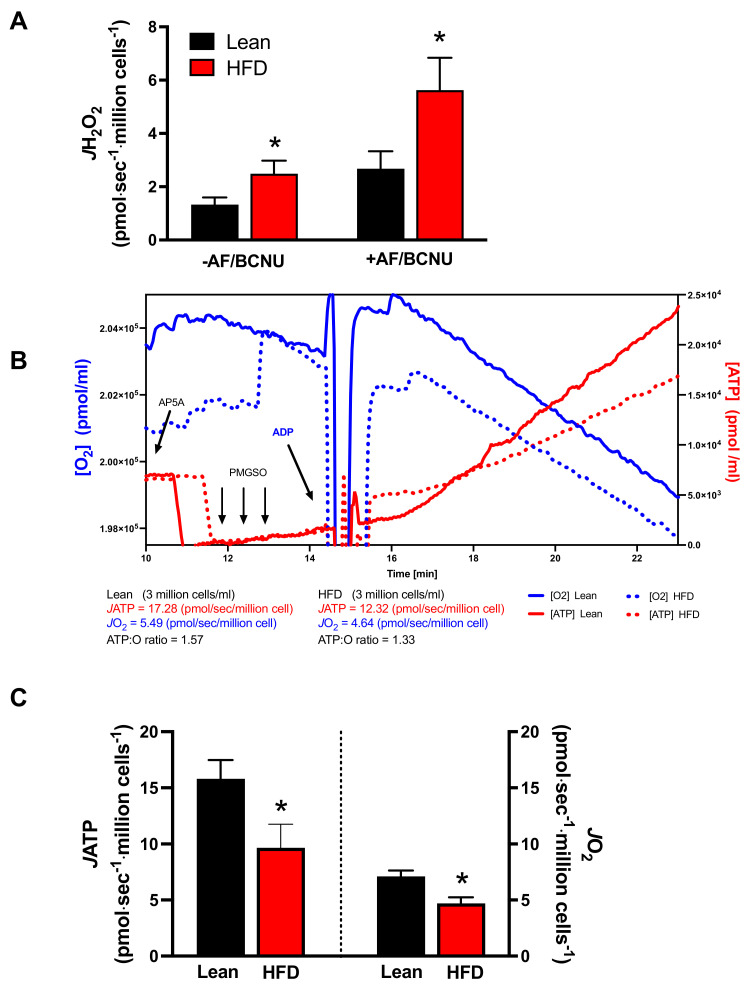
Permeabilized LPS-stimulated CD19+ B cells from HFD-induced obese mice displayed increased JH_2_O_2_ and decreased JATP and JO_2_. (**A**) JH_2_O_2_ emission (−AF/BCNU) and production (+AF/BCNU; inhibitors of thioredoxin and glutathione reductase). (**B**) Representative traces from lean and obese mice showing the simultaneous measurement of JATP and O_2_ concentration. (**C**) Summary data of JATP and JO_2_. Data are averages ± SEMs from five to seven independent experiments, * *p* < 0.05.

The next set of experiments directly investigated the mitochondrial function of B cells in response to obesity. To do so, LPS-activated B cells were permeabilized, which gave direct access to the mitochondria while preserving the three-dimensional structure of the mitochondrial network. During basal respiration supported by complex I and complex II substrates, activated B cell mitochondria from mice with obesity displayed an elevated rate of H_2_O_2_ (JH_2_O_2_) emission (Figure 2A), which reflects the balance between the amount produced and the amount reduced to water via the thioredoxin and glutathione redox buffering circuits. The addition of inhibitors to thioredoxin and glutathione reductase to directly measure production revealed an ~2.5-fold greater rate of JH_2_O_2_ with the HFD (Figure 2A). Next, to examine OXPHOS efficiency, we used a custom-designed apparatus and enzyme-linked assay system [16] to measure the JO_2_ and ATP synthesis rate (JATP; via fluorescence) simultaneously under submaximal ADP-stimulated clamped conditions (Figure 2B). Both JO_2_ and JATP were decreased in the B cells of obese mice relative to the lean controls (Figure 2C).

**Figure 3:**
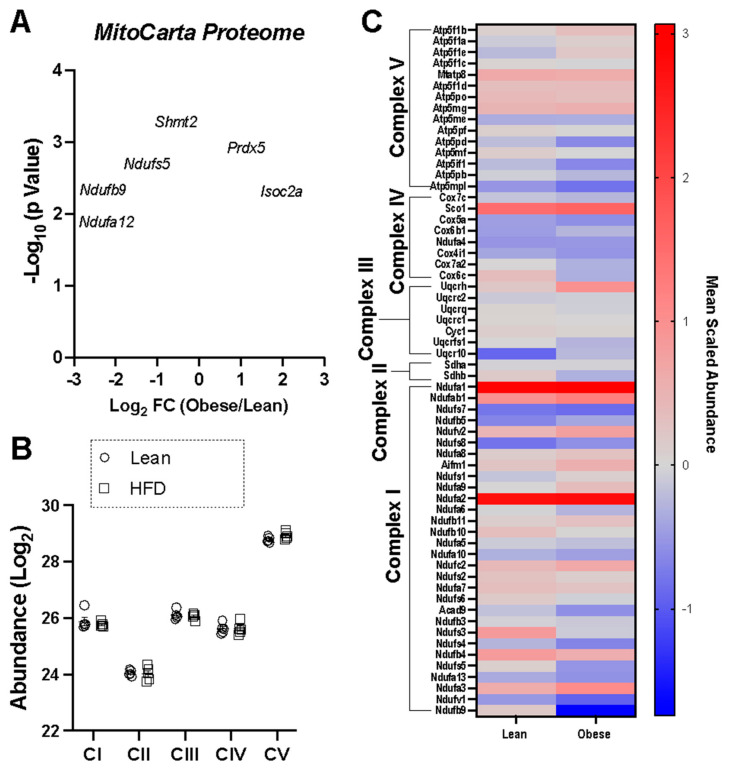
HFD-induced obesity drives a modest change in the B cell mitochondrial proteome with a notable upregulation of units of complex I. (**A**) Volcano plot of mitochondrial protein abundance in LPS-stimulated obese cells versus lean B cells. (**B**) Summed abundance of each OXPHOS complex. (**C**) Heatmap displaying the mean scaled protein abundance of the individual protein subunits of the OXPHOS complexes. The data are averages from five independent experiments.

To investigate potential mechanisms underlying the lower respiratory function in the B cells of obese mice, label-free quantitative proteomics analysis was carried out on LPS-stimulated B cells. Looking exclusively at the mitochondrial proteome, relative to lean mice, the most upregulated mitochondrial protein in the B cells of obese mice was the H_2_O_2_ detoxifying enzyme peroxiredoxin-5 (PRDX5) (Figure 3A). The summed abundance of complexes I–V was similar between the groups (Figure 3B). Three of the top ten most downregulated mitochondrial proteins in the B cells of obese mice were all accessory subunits of respiratory complex I (NDUFS5, NDUFB9, and NDUFA12), suggesting potential alterations in complex I assembly/function (Figure 3C).

**Figure 4:**
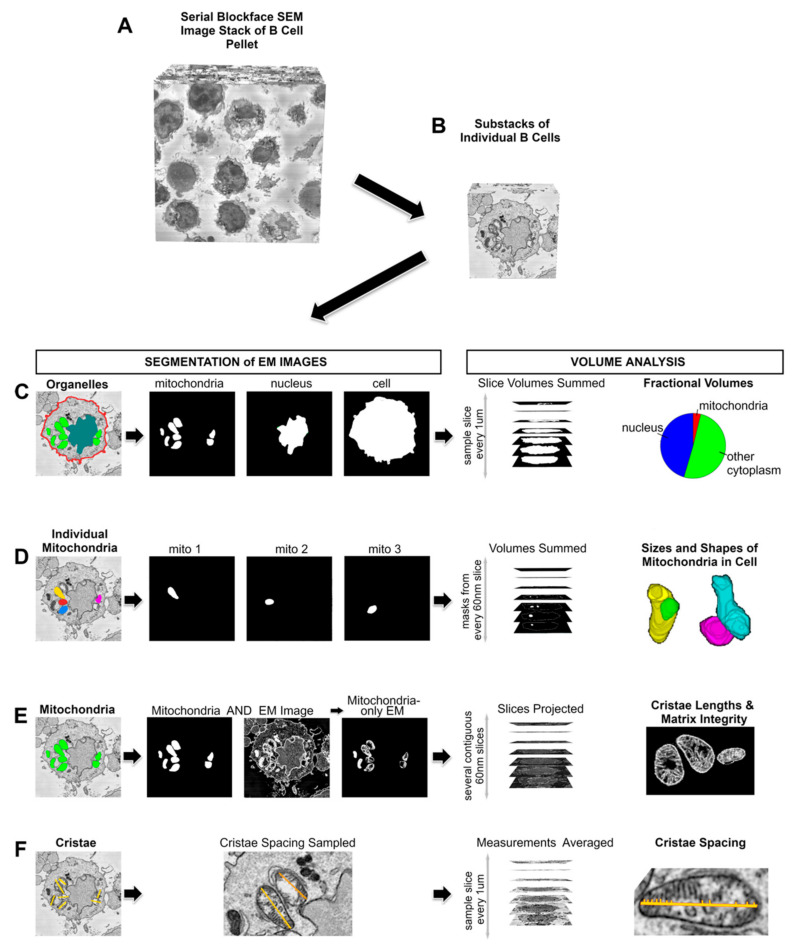
Volume EM provides a 3D profiling of the changes in B cell mitochondria. For each embedded B cell pellet, a stack of approximately 300–500 images was generated at 60–70 nm steps with 5 nm pixels (**A**). At least six cells were chosen that lay wholly within each stack, and a substack was generated for each cell (**B**). They were then used to assess fractional volumes (**C**), the characteristics of the mitochondria making up the cell network (**D**), and the cristae ultrastructure (**E**,**F**), as explained in detail in Section 2.

Finally, we used volume EM to investigate whether HFD-induced obesity promoted changes in the mitochondrial volume and cristae structure, which are important for respiratory activity. B cells from the spleens of lean and obese mice were activated with LPS for 24 h and then fixed for serial blockface EM. From each sample (four lean mice and five obese mice), serial EM image stacks of around 30 cells were generated (Figure 4A), and at least five whole cells were cropped out (Figure 4B) for analysis. Mitochondria and other cellular structures were segmented as binary masks (Figure 4C–F), and mitochondrial volume, volume per cell, mitochondrial shape, and cristae structure measurements were generated.

**Figure 5:**
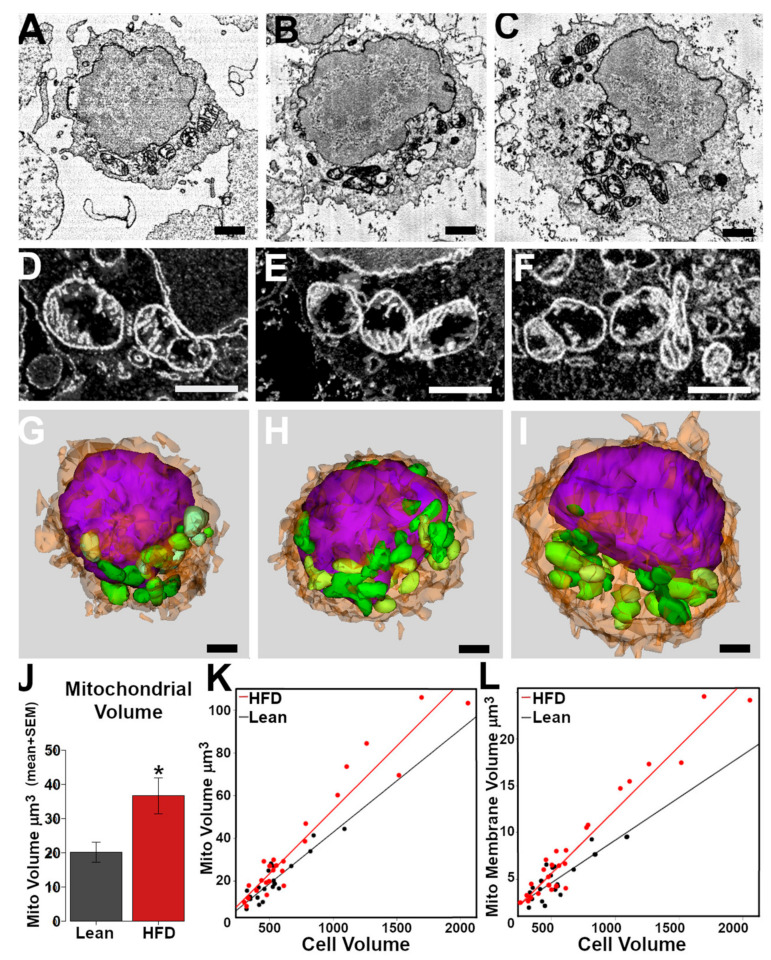
HFD-induced obesity increases the mitochondrial volume of activated B cells. B cells illustrated from lean mice (**A**,**D**,**G**); cell volume: ~800 μm^3^ and from HFD fed mice (**B**,**E**,**H**); ~800 μm^3^; (**C**,**F**,**I**); ~1300 μm^3^. (**A**–**C**) EM Image slices from the perinuclear cytoplasm indicating mitochondria (m) distribution. (**D**–**F**) Higher-magnification 3D projections of slices covering a 1 μm depth. Mitochondria from each cell consistently had regions of intact cristae (**C**) and regions lacking cristae (*). (**G**–**I**) Three-dimensional models from reconstructed cells illustrating the size and shape of mitochondria (green) adjacent to the nucleus (purple). (**J**) Average mitochondrial volume in the lean and obese groups’ B cells (* *p* < 0.033, *t*-test, *n* = 4–5 animals). (**K**) Scatter plots and regression lines for mitochondrial versus cell volume (K, (lean group; slope = 0.048, adjusted R2 = 0.81, *p* < 3.9 × 10^−8^; HFD group; slope = 0.0599, adjusted R2 = 0.926, *p* < 1.04 × 10^−14^)). (**L**) Scatter plots and regression lines for mitochondrial membranes (matrix excluded) versus cell volume (lean group: slope = 0.009, adjusted R2 = 0.654, *p* < 9.7 × 10^−6^; HFD group: slope = 0.0135, adjusted R2 = 0.939, *p* < 1.08 × 10^−15^). Scale bars: 1 μm.

We examined the mitochondrial structure in similarly sized B cells from lean (Figure 5A) and obese (Figure 5B) mice. As the B cells were larger from obese mice, we also considered whether large cells may have been differentially affected (a volume greater than 1000 µm^3^, Figure 5C). The mitochondria in both the lean and obese groups had a heterogeneous appearance. In many cases, the same mitochondrion had regions with a dense matrix and well-ordered cristae adjacent to regions that lacked cristae and had a lucid matrix (Figure 5D–F). Activated lymphocytes have been described as having this appearance [26]. The cristae membrane volumes (Figure 4C and Figure 5K) and inter-cristae spacing (Figure 4D) were similar between the groups and the small and large cells in the HFD group. A 3D analysis of individual mitochondria indicated that the mitochondrial size and shape were also similar between the groups (Figure 5G–I). Spherical mitochondria made up <10% of the mitochondria in the lean and obese groups, indicating that the results were not due to artifactual fragmentation in the tissue culture or fixation.

The average volume occupied by the mitochondria in the B cells (cellular fractional volume) from the obese mice was twice that of the lean mice (Figure 5J). However, this was not simply the result of cell size differences. Regression plots demonstrated a strong correlation between mitochondrial volume and cell volume (Figure 5L), but the regression slopes were different for the two groups, with the obese group cells having ~23% greater mitochondrial volumes than lean-group cells of the same size. We also compared the mitochondrial membrane-only volume and cell volume (Figure 5L). The membrane-only volume represents the inner and outer mitochondrial membranes and cristae membranes segmented from each mitochondrion (Figure 4E) and excludes the mitochondrial matrix or swollen areas. A strong correlation indicated that the B cells from the obese group had ~50% more mitochondrial membrane than the similar-sized cells in the lean group. These results indicate that there is a propensity for B cells of obese mice to have additional mitochondrial volume compared with similarly sized lean B cells, that their mitochondria are similar ultrastructurally, and that the increases are not due to mitochondrial distension.

## 4. Discussion

Herein, we focused on a proof of concept that mitochondria may be targets of diet-induced obesity in B cells. The rationale for studying mitochondria is that they are critical for B cell development, activation, and differentiation toward memory or plasma cell fates [27]. Therefore, it is plausible that obesity may target differing aspects of B cell mitochondrial activity that would contribute to changes in B cell differentiation and activation that would ultimately impair inflammatory, metabolic, and infectious responses.

A significant advancement of this work is that HFD-induced obesity decreased B cell mitochondrial respiratory function, as measured with intact cells, which was further confirmed with simultaneous measures of JO_2_ and JATP using permeabilized cells. The HFD also elevated the rate of mitochondrial H_2_O_2_, which is linked to impaired humoral immunity [28]. The increase in H_2_O_2_ is intriguing, as B cell mitochondrial respiratory activity is directly tied to the activity of specific transcription factors. For example, the transcription factor BLIMP1 reduces B cell mitochondrial mass in B cells, which lowers ROS and thereby inhibits class-switch recombination to drive plasma cell formation [29]. We have previously reported that BLIMP1 levels are lowered with obesity in the bone marrow and that the percentage and number of CD138+ plasma cells in obesity are also decreased [15]. This suggests future studies on how a reduction in BLIMP1 levels may be leading to increased levels of H_2_O_2_ and thereby decreasing the formation of antibody-secreting plasma cells. Overall, our results set the basis for future experiments on how obesity may target B cell differentiation through B cell ROS production.

The data from this study were generally consistent with the emerging literature suggesting that obesity impairs lymphocyte metabolism. To exemplify, there are data to suggest that obesity impairs the metabolic phenotype of B cells. One study demonstrated that adults with obesity display a B cell hyper-metabolic phenotype in white adipose tissue compared to B cells in peripheral blood [30]. It has been hypothesized that the increase in bioenergetic demand within B cells localized to white adipose tissue is driven by the need to secrete autoimmune antibodies [31]. Our results are also consistent with previous reports that obesity can target the metabolic phenotype of T cells [32]. For example, CD4+ T cells isolated from obese mice displayed increased glucose uptake and an increased oxygen consumption rate, which was reversed in a culture with metformin [33]. We have also observed impaired glycolytic and mitochondrial metabolism with pulmonary CD8+ T cells from influenza-infected obese mice [32].

To identify mechanistic targets, we first focused on how HFD-induced obesity could control the B cell proteome. Interestingly, we found a significant upregulation with PRDX5, which is consistent with the notion that the cells of individuals with obesity try to deal with excess H_2_O_2_. Furthermore, we found a downregulation of the accessory subunits of complex I. These results open the door to future studies on how obesity may impact complex I assembly and, moreover, the formation of supercomplexes. There is evidence that, in human subjects with type 2 diabetes, complex I levels and supercomplex formation are lowered in the hepatic tissue, and there may be a parallel in human B cells, which will require further investigation [34].

This study’s image analyses also paves the way for future experiments. We observed tremendous heterogeneity in B cell mitochondria sizes and shapes, consistent with the literature on other cell types in the context of cellular heterogeneity [35]. Although the implications of an increased mitochondrial volume with obesity are not clear, these results warrant further investigation into how obesity may target differing aspects of mitochondria structure–function. We have previously demonstrated that metabolic overload and changes in diet composition can lead to significant remodeling of the mitochondrial inner mitochondrial membrane bilayer and, thereby, its structural properties in metabolically active organs, such as the heart [36,37]. Perhaps similar changes occur within the mitochondria of differing B cell populations, which remains to be investigated. Furthermore, the data suggest that the overall increase in the total mitochondrial volume per cell is due to increased mitochondrial biosynthesis or reduced mitophagy and not simply swelling of the mitochondria; moreover, obesity may also target the fusion/fission equilibrium [38]. The functional significance of these changes remains to be established.

There are several limitations to this study. First, we did not study B cells that were naive, which was driven by technical limitations. We initially attempted to measure the respiratory function of naive B cells using a high-resolution respirometer, but we did not have a sufficient signal to obtain reproducible results. Thus, future studies will require an extensive pooling of mice and/or the use of other approaches to study the B cell mitochondrial respiratory activity of naive B cells. Second, we did not isolate differing B cell populations, such as antibody-secreting cells or memory B cells. We also did not tease apart marginal zone B cells that express high levels of Toll-like receptor 4 (TLR4) and bind LPS compared to other subsets that are low in TLR4 expression. Third, our experiments were conducted with male mice. We have previously demonstrated sex differences in the B cell response using cells isolated from obese mice and humans with obesity [15]. The rationale for focusing on males was that female mice are not as metabolically impaired as males [15].

A final limitation is that we did not connect changes in mitochondrial respiratory activity in response to HFD-induced obesity with metabolic and immunological outcomes. For instance, future studies will need to connect how the observed changes with B cell respiratory activity impact glutamine, pyruvate, and fatty acid metabolism. In addition, it would be of utility to measure B cell activation and other functional outcomes. We previously demonstrated that activated splenic B cells isolated from obese mice secreted less IgM and IgG relative to naive controls [39]. Others have also shown that B cell functional responses and phenotypes are dysregulated with obesity or obesity-related complications relative to controls [7,10,40,41,42,43,44,45,46]. The objective here was to establish a proof of concept that sets the stage for future experiments focused on how obesity impairs mitochondrial respiratory activity of specific B cell subsets to impair humoral immunity and drive inflammation and insulin resistance.

## 5. Conclusions

In summary, our data show with the use high-resolution respirometry that activated B cells from HFD-induced obese mice, relative to controls, have lower ATP-linked and maximal uncoupled respiration. In addition, activated B cells of obese mice had increased H_2_O_2_ emission and production and decreased JO_2_ and JATP relative to controls. Proteomic and electron microscopy analyses revealed potential underlying mechanistic targets of HFD-induced obesity on B cells. These studies set the basis for subsequent studies on how obesity impacts B cell bioenergetic metabolism and, ultimately, immunological function.

## Data Availability

The study’s data are contained within the article.

## Supplementary Materials

The following supporting information can be downloaded at: https://www.mdpi.com/article/10.3390/nu15224807/s1, Figure S1: Metabolic profile of C57BL/6J mice fed a high fat diet. (A) Body weights in response to a control lean diet or high fat diet for C57BL/6J male mice. (B) Glucose and (C) insulin levels from an IPGTT conducted after a 5 h fast. In B, glucose levels were measured 15 min prior to administration of glucose at time 0. Data are average ± SEM for *n* = 12 mice per group for body weight and *n* = 4–6 for IPGTT per group. * *p* < 0.05.
