# Supplementary material for: High Fat Diet-Induced Obesity Dysregulates Splenic B Cell Mitochondrial Activity
Source: Nutrients. 2023 Nov 17;15(22):4807. doi: 10.3390/nu15224807 (PMC10675399; doi:10.3390/nu15224807)
Supplement: Supplementary file 1 [file nutrients-15-04807-s001.zip › nutrients-2693776-supplementary.pdf]

High Fat Diet-Induced Obesity Dysregulates Splenic B Cell Mitochondrial Activity

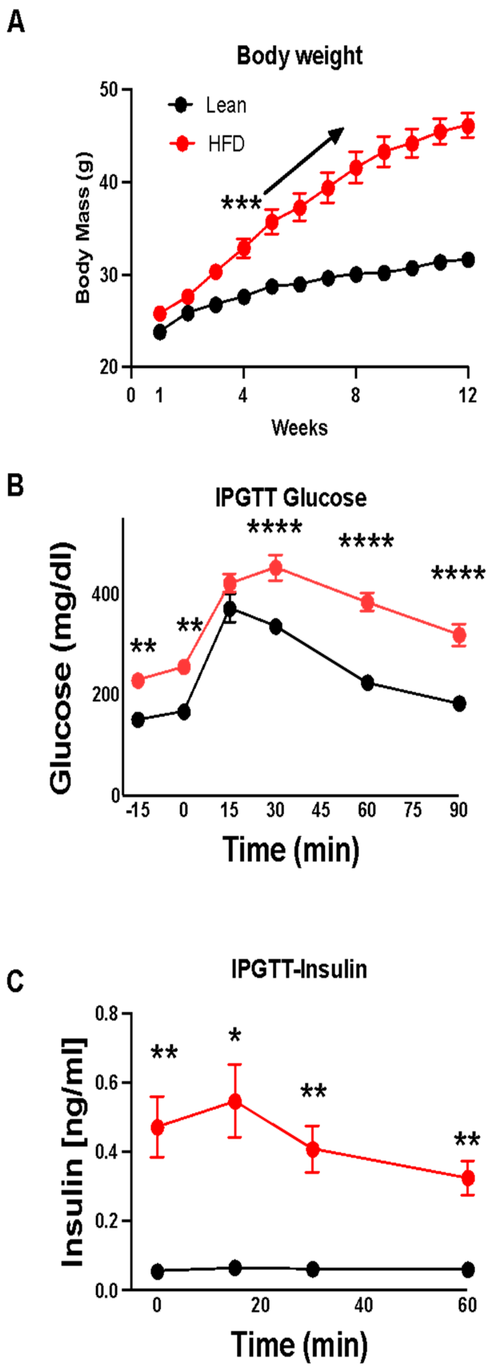

Figure S1. Metabolic profile of C57BL/6J mice fed a high fat diet. (**A**) Body weights in response to a control lean diet or high fat diet for C57BL/6J male mice. (**B**) Glucose and (**C**) insulin levels from an IPGTT conducted after a 5 h fast. In B, glucose levels were measured 15 min prior to administration of glucose at time 0. Data are average ± SEM for $n = 12$ mice per group for body weight and $n = 4$–6 for IPGTT per group. * $p < 0.05$.
